# The Arabidopsis Mei2 homologue AML1 binds AtRaptor1B, the plant homologue of a major regulator of eukaryotic cell growth

**DOI:** 10.1186/1471-2229-5-2

**Published:** 2005-02-21

**Authors:** Garrett H Anderson, Maureen R Hanson

**Affiliations:** 1 Molecular Biology and Genetics, Cornell University, Ithaca, NY 14850, USA

## Abstract

**Background:**

TOR, the target of the antibiotic rapamycin in both yeast and mammalian cells, is a potent cell growth regulator in all eukaryotes. It acts through the phosphorylation of downstream effectors that are recruited to it by the binding partner Raptor. In Arabidopsis, Raptor activity is essential for postembryonic growth. Though comparative studies suggest potential downstream effectors, no Raptor binding partners have been described in plants.

**Results:**

AtRaptor1B, a plant Raptor homologue, binds the AML1 (Arabidopsis Mei2-like 1) protein in a yeast two-hybrid assay. This interaction is mediated by the N-terminal 219 residues of AML1, and marks AML1 as a candidate AtTOR kinase substrate in plants. The AML1 N-terminus additionally carries transcriptional activation domain activity. Plants homozygous for insertion alleles at the *AML1 *locus, as well as plants homozygous for insertion alleles at all five loci in the *AML *gene family, bolt earlier than wild-type plants.

**Conclusion:**

AML1 interacts with AtRaptor1B, homologue of a protein that recruits substrates for phosphorylation by the major cell-growth regulator TOR. Identification of AML1 as a putative downstream effector of TOR gives valuable insights into the plant-specific mode of action of this critical growth regulator.

## Background

TOR, the target of the antibiotic rapamycin in both yeast and mammalian cells, is a major regulator of cell growth and translation [1]. TOR is a large (over 2,400 residues) protein kinase [2] present in all eukaryotes analyzed. It is thought to act in a nutrient-sensitive complex TORC1 with Raptor (regulatory associated protein of TOR) and another protein to regulate cell growth [3-7] – though there is some debate on the nutrient sensitivity of the complex [8]. Raptor, a protein with HEAT and WD-40 protein interaction domains, recruits substrates for phosphorylation by TOR in yeast and mammals [3,7]. TOR also acts in a second, nutrient-insensitive complex without Raptor to regulate the cytoskeleton [3,8,10].

Disruption of the Arabidopsis TOR homologue AtTOR is lethal early in plant embryonic development [11]. Disruption of AtRaptor (encoded by two paralogous loci in Arabidopsis) causes seedling developmental arrest but allows normal embryonic development [[12], manuscript in preparation], suggesting that AtTOR embryonic activity is independent of AtRaptor and that the TOR-Raptor complex has been adapted in the ancestor of the angiosperms to regulate post-embryonic growth. In support of this, AtTOR has been show to be expressed in dividing and expanding cells [11]. Thus, identifying downstream activators of TOR signaling may provide insights into the activity of AtTOR in post-embryonic growth.

Mei2 is a putative TOR substrate and potent meiosis-signaling molecule identified in the fission yeast *Schizosaccharomyces pombe *[13,14]. Mei2 is bound by the Raptor homologue Mip1 [15], and is an inactive phosphoprotein under high nutrient conditions [16,17] – conditions which promote TOR kinase activity. The kinase governing two of the three Mei2 phosphorylation sites is known [16]; TOR is a strong candidate for the kinase governing the third. In diploids under low nutrient conditions, unphosphorylated Mei2 accumulates and localizes to the nucleus, where it binds to a noncoding, mRNA-like RNA molecule meiRNA in an interaction mediated by the third of its three RNA Recognition Motifs (RRMs)[14,16,18]. The Mei2-meiRNA interaction occurs as meiRNA is being transcribed, tethering Mei2 to the meiRNA locus [19]. Accumulation of Mei2 at this focused point immediately precedes meiosis.

Mei2-like proteins are predicted in a wide range of organisms [20,21] including some fungi, alveolates, a diatom, and all land plants, but they are absent from metazoans and budding yeast. In land plants, predicted Mei2-like proteins form a small conserved gene family, many of whose members' transcripts accumulate in the shoot apical meristem specifically or in the shoot apical meristem in addition to a range of mature tissues [20].

Arabidopsis Mei2-like 1 (AML1), the first member of this family to be described, was isolated in a screen for plant cDNAs whose expression could complement defects in the fission yeast meiosis signaling pathway [22]. Like Mei2, AML1 has three RRMs. Expression of a protein fragment containing only the third AML1 RRM was sufficient for restoration of meiosis signaling in fission yeast lines with defects upstream of Mei2, but not in lesions of the *mei2 *locus itself. Terminal Ear 1 (te1), a more divergent member of the *mei2*-like family of genes, regulates leaf initiation in maize; the tassel of mutant plants is encased a whorl of leaves superficially resembling a maize ear [23].

Given the potential role of downstream AtTOR effectors in post-embryonic growth, the intriguing signaling activity of Mei2 in fission yeast, and the known interaction between Mei2 and the fission yeast Raptor protein, we asked if the Mei2-Raptor interaction was conserved between its Arabidopsis orthologues AML1 and AtRaptor1B.

## Results

### AML1 and AtRaptor1B interact in a yeast two-hybrid assay

To test for an interaction between the Arabidopsis proteins AML1 and AtRaptor1B, the open reading frames (ORFs) of each of the transcripts predicted to encode these proteins were amplified via PCR with primers carrying suitable restriction sites for cloning into the yeast two hybrid vectors pGADT7 and pGBKT7. pGADT7 encodes a transcriptional activation domain (AD) which can recruit the yeast transcription machinery. pGBKT7 encodes a DNA binding-domain (BD) which binds to the promoters of *ADE2 *and *HIS3*. pGADT7 and pGBKT7 additionally carry the genes *LEU2 *and *TRP1*. When transformed into the yeast line AH109, which is *leu2*^-^*trp1*^-^*ade2*^-^*his3*^-^, co-transformed cells will grow on yeast medium lacking leucine and tryptophan. Cells co-transformed with two-hybrid constructs encoding proteins that interact will grow on media additionally lacking histidine and adenine (selective media).

AH109 yeast cells co-transformed with clones encoding AML1 and AtRaptor1B in complementary two-hybrid vectors grew on selective media. Co-transformations of control pGBKT7 and pGADT7 empty vectors, or either control vector co-transformed with its complement harboring the AML1 or AtRaptor1B ORF, yielded cells able to grow on media lacking leucine and tryptophan but not on selective media.

Evidence from other systems indicates that Raptor protein fragments lose the ability to bind substrates [4]. Therefore, only full length AtRaptor1B was tested in this assay. Mei2, the fission yeast AML1 homologue, is highly modular. It is divided into distinct N-terminal and C-terminal domains. The N-terminal half of the protein appears to play a regulatory role [24]. The C-terminal half of Mei2 is sufficient to complement lesions in the *mei2 *locus [16]. Additionally, the AML1 C-terminal half, expressed in fission yeast meiosis signaling mutants, is able to complement meiosis signaling defects upstream of Mei2 [22]. Therefore we generated clones encoding fragments of AML1 and assayed them for interactions with full length AtRaptor1B.

**Figure 1:**
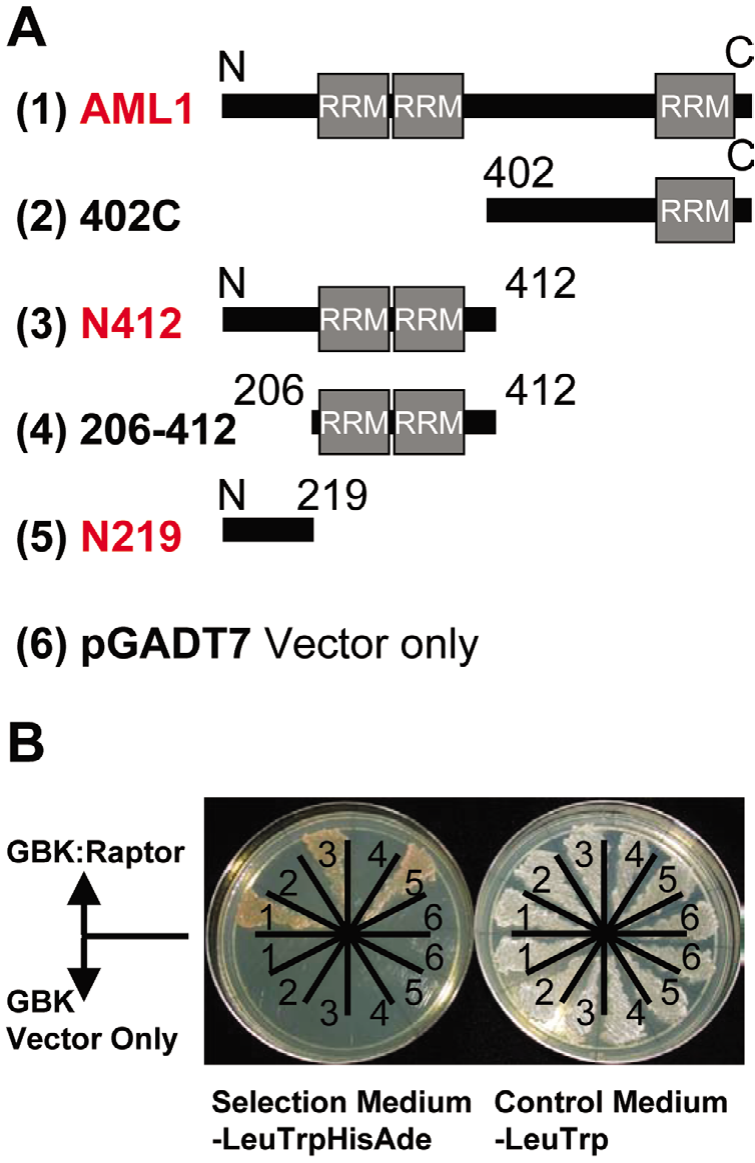
**AML1 interacts with AtRaptor1B in a yeast two-hybrid assay**. A. Schematic diagram of AML1 fragments cloned into pGADT7. The AML1 RNA Recognition Motifs (RRMs) are indicated. Fragments interacting with AtRaptor1B are labeled in red. B. Yeast two-hybrid results. Numbers on each plate refer to the pGAD construct in A. For each plate, the set of six cultures on the top half were co-transformed with pGBK:Raptor; the set of six cultures on the bottom half were co-transformed with pGBKT7 vector. The plate at left lacks leucine, tryptophan, histidine and adenine and selects for a protein-protein interaction. The control plate at right lacks leucine and tryptophan, and indicates co-transformation of the yeast cells with both a pGAD and a pGBK plasmid.

AML1 fragments N412 and N219, comprised of the first 412 or the first 219 residues of the 915 residue AML1 protein, restored growth on media lacking leucine, tryptophan, histidine and adenine when cloned into pGADT7 and co-transformed with pGBK-Raptor (Fig. 1). Neither N163, nor 155–219, (the fragments which together comprise N219), nor 155–412 (which with N163 comprises N412), could restore growth when cloned into pGADT7 and co-transformed with pGBK-Raptor. AML1 fragment 402C, comprised of residues 402 to the C-terminus of the protein, and all C-terminal fragments tested (695C, 402–704) failed to restore growth on selective medium. None of the AML1 fragments cloned into pGADT7, and cotransformed with empty pGBKT7 control vector, yielded transformants able to grow on selective medium.

### AML1 fragments N412 and N163 harbor activation domain activity in a yeast one-hybrid assay

AML1 N412, cloned into pGBKT7, restored growth on selective medium to cells co-transformed with either pGAD:Raptor or pGADT7. To investigate this result further, we tested all AML1 fragments for native transcriptional activation domain activity in a yeast one-hybrid assay. AML1 fragments were cloned into pGBKT7, singly transformed into AH109 yeast cells, and assayed for growth on media lacking tryptophan (to confirm transformation) and media additionally lacking adenine. The DNA binding-domain of the pGBKT7 tethers any C-terminally fused fragments to the *ADE2 *promoter. In the absence of a binding partner, BD-fusion chimeric proteins trigger transcription of *ADE2 *only if the protein fused to the BD contains native transcriptional activation activity.

**Figure 2:**
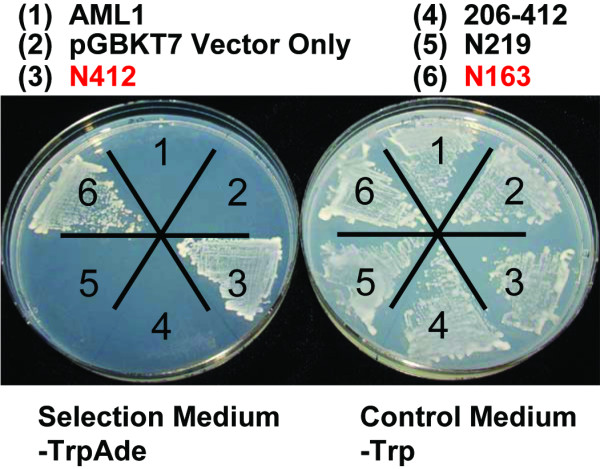
**The AML1 N-terminus harbors transcriptional activation domain activity. **One-hybrid assay on AML1 fragments cloned into pGBK. The plate at left lacks tryptophan and adenine, and growth indicates transcriptional activation domain activity in the tested fragments. The control plate at right lacks adenine, and indicates transformation of the cells with the desired pGBK construct.

AML1 N412 and AML1 N163, but not full length AML1, AML1 N219 or any C-terminal AML1 fragments, were able to restore growth on media lacking tryptophan and adenine (Fig. 2). AML1 N122 was similarly unable to restore growth. AML1 fragments 155–219 and 155–412, in pGBKT7, could not be stably transformed into yeast. These results were observed in multiple independent transformant lines for a given construct. Cotransformation of any of the pGBKT7-derived constructs with pGADT7 did not affect the growth of any of the transformed lines on media lacking tryptophan and adenine.

### Plants homozygous for insertion alleles of *AML1 *and of all five *AML *family members show early flowering

**Figure 3:**
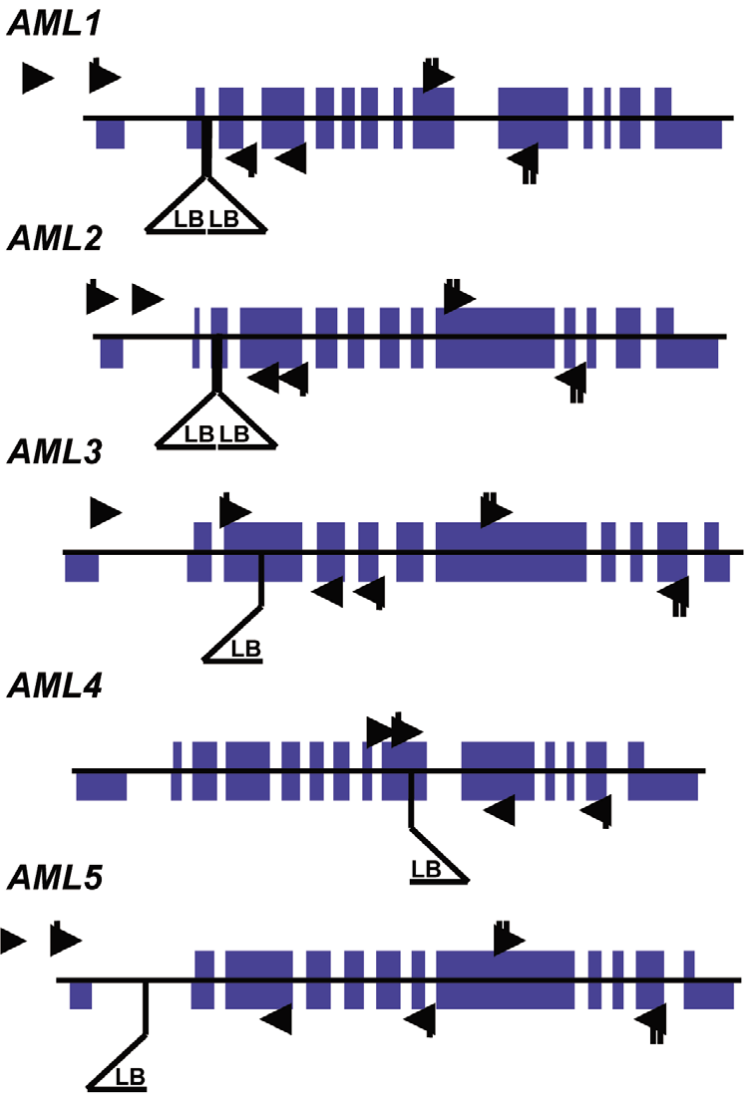
***AML *loci and insertion alleles. **The position of the insertion in each locus is indicated by a triangle below the locus picture. The wild-type loci were described previously (17). The thin central line indicates genomic DNA. Solid blue blocks spanning the central line indicate coding exons. Solid blue blocks below the line indicate untranslated regions. T-DNA insertion left borders are indicated with a triangle below the locus. Binding sites for primers used to genotype the wild-type allele of each locus are indicated with arrows. Binding sites for primers used to ascertain the effect of homozygosity at the mutant allele are indicated with triangles tagged by a single hash line if the PCR product spans the insertion site, or a doublet hash line if the PCR product is from a region downstream of the insertion site.

An interaction with AtRaptor1B points to AML1 as a downstream effector of TOR signaling in plants. Additionally, the dramatic phenotype *mei2 *disruption and the intriguing mode of Mei2 action led us to ask what the consequences would be of disruption of the *AML1 *locus and of all five *AML *gene family members. To obtain insertion alleles in *AML1 *and other *AML *gene family members, we screened the insertion allele populations at the University of Wisconsin Arabidopsis Knockout facility [25], obtaining alleles harboring insertions in *AML1*, *AML3*, *AML4 *and *AML5 *(Fig. 3). An *AML2 *insertion allele was obtained from the SIGnAL collection at the Salk Institute [26].

**Figure 4:**
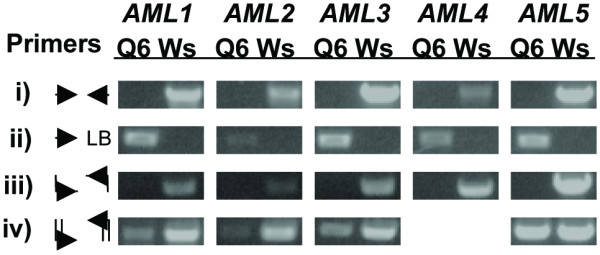
***AML *mutant allele characterization. **i) PCR product to identify the wild-type allele of each *AML *locus. ii) PCR product to identify the mutant allele of each *AML *locus. iii) PCR product to assay for accumulation of cDNA from transcripts transcribed across the insertion site. iv) PCR product to assay for accumulation of cDNA from transcripts transcribed from the AML locus downstream of the insertion site. AML4 was excluded from this assay because the mutant allele is disrupted in the center of its coding region. Q6 refers to genomic DNA or cDNA template extracted from quintuple insertion allele homozygotes; Ws refers to genomic DNA or cDNA template extracted from wild-type (Wassilijewskia ecotype) plants.

By RT-PCR using primers which anneal to the cDNA at sites spanning the insertion site of each insertion allele, we established that no wild-type transcripts accumulate in homozygous mutants (Fig. 4). By a series of crosses, we then generated higher-order insertion homozygotes, culminating in the quintuple insertion homozygotes Q6 and Q17. All lines were viable and fertile.

**Figure 5:**
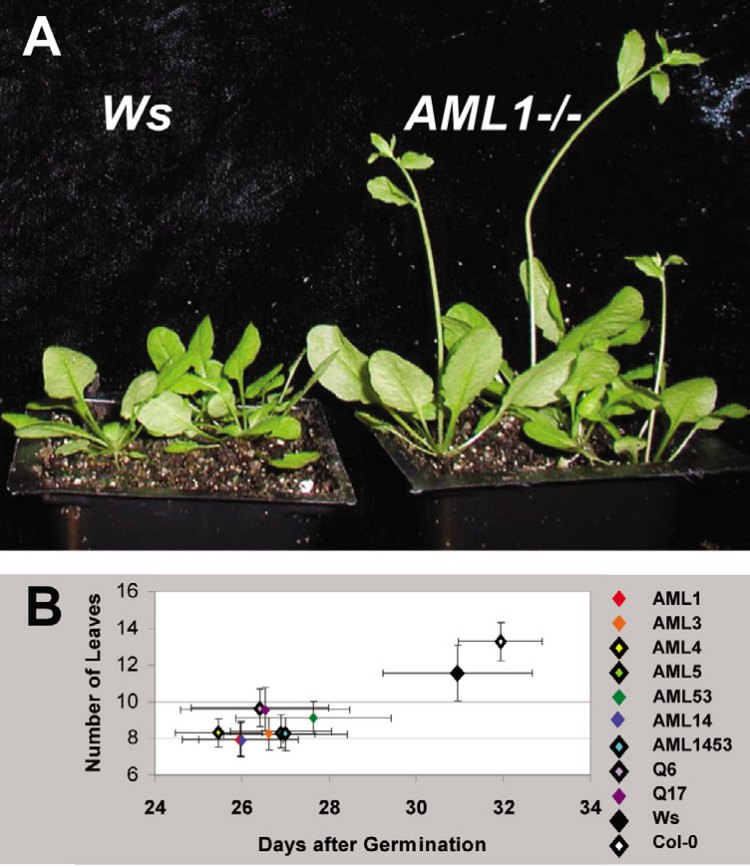
***AML *insertion mutants bolt early. **A. Wassilijewskia (Ws) and AML1 insertion homozygotes (*AML1*-/-) grown under long days. B. Bolting time and number of rosette leaves at time of bolting for AML single insertion and higher order insertion mutants. Plants were grown under 16 hour days. Ws and Columbia (Col 0) ecotypes are the two points in the top left of the graph; the cluster of points at the lower right are all *AML*-/- mutants.

AML insertion homozygotes bolted earlier than wild-type lines (Fig. 5A and 5B). This effect was independent of the number of insertion alleles carried by the mutants; AML 'quint' lines Q6 and Q17 were not qualitatively different than lower order insertion allele homozygotes.

Additionally, AML insertion homozygote seedlings were assayed for a differential response to a range of signaling molecules. Seedlings were germinated on culture medium supplemented with the gibberellic acid GA_3_, paclobutrazol, the auxin 2,4-D, 1-amino-cyclopropane-1-carboxylic acid, 1% sucrose, 6% sucrose and kinetin, and in the dark. Quintuple insertion mutants responded slightly more than wild-type seedlings to GA_3 _as measured by the change in seedling length in the presence vs. in the absence of the hormone. This effect was repeatable but weak, and no other differential hormonal response was observed (data not shown).

### Sequence downstream of the mutant allele insertion sites is transcribed

Given the mild phenotype of the AML insertion homozygotes, we further investigated the extent of the effect of the insertions at each locus. RT-PCR, as previously stated, showed that no wild-type transcripts accumulate in lines Q6 and Q17. In fission yeast, however, the C-terminal half of the protein is sufficient to complement lesions of the *mei2 *locus [16]. The insertion alleles of all but *AML4 *are disrupted at or near the 5' ends of their predicted coding regions. We therefore designed primers that anneal to the region downstream of the insertion site in each allele and performed polymerase chain reactions to assay for accumulation of fragments capable of encoding the C-terminal half of any of the AML proteins (Fig. 4). To determine whether the amplified fragments corresponded to *AML *cDNAs, we performed restriction digests on the PCR products, which confirmed that the cDNAs originated from *AML *gene transcripts. Weak amplification of cDNA representing transcripts originating downstream of the insertion site was observed for AML1, AML2 and AML3; amplification of the AML5 3' region was indistinguishable from the amplification seen from of wild-type cDNA template.

### Transgenic lines overexpressing AML1:GFP or GFP:AML1 fusion proteins could not be recovered

The *AML1 *ORF was separately cloned into the pCambia1302 35S::GFP plant transformation vector both 5' and 3' of the *GFP *ORF, and the construct was transformed into Arabidopsis via *Agrobacterium*-mediated floral dip [27]. Transformants, identified by resistance to the antibiotic hygromycin and confirmed through PCR, were recovered at a very low rate of less than .01%. No fluorescence was observed in any tissues of any transformants assayed, and *AML1:GFP *transcripts could not be detected via RT-PCR performed on cDNA transcribed from RNA extracted from bulk shoot tissue (data not shown).

## Discussion

Raptor proteins in yeast and mammals function by recruiting substrates for TOR, a central regulator of cell growth in response to nutrients [4,7,28]. An interaction with Raptor therefore strongly suggests that a given protein is a TOR substrate and downstream effector of TOR signaling. Plants homozygous for lesions at both *AtRaptor *loci show normal embryonic development but are unable to maintain shoot meristem activity [12]. TOR substrates, then, may play a role in regulating meristem-driven post-embryonic growth. The interaction between AML1 and AtRaptor1B implicates the AML family of proteins in TOR signaling. It points specifically to a role for the AML proteins in regulation of shoot meristem activity.

An interaction between Mei2 and the fission yeast Raptor homologue Mip1 has been reported previously; indeed, Mip1 (Mei2 interacting protein 1) was the first Raptor homologue characterized in any eukaryote [15]. The conservation of this interaction from fission yeast to plants suggests that the well-characterized Mei2 signaling pathway may provide insight into the function of the AMLs.

Mei2 is a potent meiosis-signaling molecule. It triggers pre-meiotic cell differentiation and meiosis in response to nutrient stress [13,14]. Meiosis signaling in fission yeast is a model for cell differentiation in response to external nutrient cues. Thus the AMLs may also play a role in cellular differentiation or in meiosis signaling.

Aside from the effect of Mei2 in development, there is the intriguing issue of its mode of action. Mei2 sub-cellular localization is mediated by an interaction with a noncoding, mRNA-like molecule [14,19]. There is a fairly large population of mRNA-like transcripts conserved among land plants despite lacking large conserved open reading frames [29]. Of these, the conserved alfalfa transcript *ENOD40 *has been shown to mediate the sub-cellular localization of an RNA-binding protein [30] and to mediate phytohormone responses [31]. AML1 may be a binding partner of one or more of these mRNA-like noncoding molecules in plants.

The transcriptional activation activity of the AML1 N-terminus observed in the yeast one hybrid assay has not been ascribed to Mei2 and may represent a novel activity of plant Mei2-like proteins. This activation activity localizes to the N-terminal 163 residues, but is strongly influenced by the adjacent residues. Activity is lost in N219, regained on N412 and lost again in full length AML1. This suggests that the AML1 N-terminal half has multiple configurations, and that the accessibility of the activation domain varies among configurations. We should emphasize, however, that the AML1 N-terminal fragment transcriptional activation activity has yet to be shown *in planta*, and that the activity may result from fusion to the DNA binding domain rather than being present in native AML1.

AtRaptor1B binding to AML1 is also localized to the N-terminus, and appears to be mediated by multiple sites in this region. This suggests that the N-terminus may contain a TOR phosphorylation site, and that this site may influence the configuration of the N-terminus.

The repeated failure to recover transgenic lines expressing AML1 suggests that its unregulated overexpression is lethal. Future efforts to characterize the AML proteins in transgenic plants may benefit from the use of inducible promoters driving transgene expression to circumvent the putative lethality of unregulated AML expression.

Disruption of any of the AML loci causes early bolting in plants grown under long days. However, lines homozygous for insertions in all five AML loci did not differ dramatically from lower-order insertion homozygotes, despite the fact that RT-PCR performed with primers spanning the insertion sites show that the wild-type transcript does not accumulate.

Transcripts originating downstream of the insertion sites but still capable of encoding the C-terminal half of the wild-type protein accumulate from all loci but *AML4*. This raises the possibility that the *AML *quintuple insertion homozygote lines do not represent total disruption of AML activity. Four of the five *AML *open reading frames in the insertion mutant are apparently truncated and all are divorced from their native promoters, but some promoter activity (perhaps from the 35S viral promoters harbored within the inserted DNA) remains and may be sufficient to cause transcription of AML coding region DNA downstream of the insertion site.

Viewed in this light, the early flowering phenotype of AML insertion homozygotes may arise not from the total disruption of AML activity but from the accumulation of AML proteins which, due to the truncations in their N-termini caused by the insertions, are no longer bound by AtRaptor1B, no longer phosphorylated by AtTOR, or no longer able to activate transcription of floral repressors.

Finally, these results provide a cautionary tale. RT-PCR performed using primers which span an insertion site may not be sufficient to conclude that all activity of a protein of interest is abolished.

## Conclusion

TOR is a major regulator of cell growth in eukaryotes, but little is known about its downstream effectors in plants. This work shows that AML1 binds AtRaptor1B, and suggests that the AML protein family may be phosphorylated by AtTOR in an AtRaptor1B-mediated interaction. The interaction with AtRaptor1B implicates AML1 as a downstream effector of AtTOR kinase signaling, and provides insight into the mode of action of this critical growth regulator.

## Methods

### Generating the two-hybrid constructs

*AML1 *was cloned via RT-PCR. The cDNA template was reverse-transcribed using Omniscript (Qiagen) from RNA extracted from bulk shoot tissue using Trizol Reagent (Invitrogen). Restriction sites *Nco*I and *Xma*I/*Sma*I were added to the 5' and 3' ends of the ORF and of all smaller AML1 fragments via PCR using ExTaq high-fidelity polymerase (Takara). An EST clone (RZL03b06) tagging *AtRaptor1B *was obtained from Kazusa DNA institute and sequenced. Restriction sites *Nco*I and *Eco*RI were added at the 5' and 3' ends of the ORF via PCR. pGBKT7 and pGADT7 are distributed by BDBiosciences.

### Yeast two-hybrid assay

AH109 cells (*leu2*^-^*trp1*^-^*ade2*^-^*his3*^-^) were grown in YEPDA liquid plates or on YEPDA plates with 17 g/L Agar-Y (Bio101 Systems). Cells were transformed using the Yeastmaker Yeast Transformation System2 (BD Biosciences) and plated on medium lacking the appropriate macronutrients (Bio101 Systems). Colonies were observed 3–7 days after transformation.

### Genotyping of insertion alleles

DNA from lines harboring insertion alleles was extracted using the alkaline boiling method [32]. Provisional homozygotes were confirmed via a second extraction using the C-TAB DNA extraction protocol. PCR to assay for wild-type and insertion alleles was performed in 20 uL volumes using ExTaq polymerase and buffers and the following cycling parameters: 94°C, 15 seconds; 61°C, 30 seconds; 72°C, 2 minutes; 35 cycles. Genotyping primers were as follows: AML1-5sm 5'atagaaggaaacaaaaaggaaaggaggaa3'; AML1-3sm 5'tagcatatcacttccctgtagccgcactg3'; AML2-5sm 5'attgctctgtctctgatgatgttttgtcg3'; AML2-3sm 5'gcagcaatatttctaaagcatcgggttca3'; AML3-5sm 5'ctttagttccctctttcctctgctgtgat3'; AML3-3sm 5'ctgccaagaacgggaaaacaaacataaa3'; AML4-5sm 5'ttgcaagcggtagtccatataaatcctc3'; AML4-3sm 5'atgctaccgggagaacctaagtgaaatc3'; AML5-5sm 5'tctttagccacatcaatcattctcatcct3'; AML5-3sm 5'atcagcgtcaagttccattcctcctccac3'; JL-202 5'cattttataataacgctgcggacatctac3'; JL-270 5'tttctccatattgaccatcatactcattg3'; pROC-737 5'gggaattcactggccgtcgttttacaa3'. The wild-type loci were assayed with the above pairs. The insert was assayed using the following pairs: AML1-5sm or AML1-3sm with JL-270 or JL-202, AML2-5sm with pROC-737, AML3-5sm with JL-270 or JL-202, AML4-3sm with JL-270 or JL-202, AML5-5sm with JL-202.

Insertions in the *AML1 *and *AML5 *loci were obtained from the University of Wisconsin alpha collection using their described protocol and are in the Wassiljewskia (Ws) ecotype background. The AML1 insertion was found in pool CSJ8-46-H35. The AML5 insertion was found in pool CSJ1091-H45. Insertions in the *AML3 *and *AML4 *loci were obtained from the University of Wisconsin Basta collection and are in the Ws background. The *AML3 *insertion was found in pool 67-6-F. The *AML4 *insertion was found in pool 18-2-H. The insertion in *AML2 *was obtained from the line 029713 from the Salk collection and is in the Columbia (Col-0) ecotype background. Aside from regions genetically linked to any of the insertion loci, the *AML *quintuple insertion allele homozygote lines are in the genomic background of a Col-0 × WS F_3 _individual once backcrossed to the WS background.

### RNA extraction, RT-PCR

RNA extraction was performed using TRIzol™ Reagent (Invitrogen) essentially according to manufacturer's instructions. Total RNA was treated with DNA-*free*™ DNase (Ambion) and reverse transcribed using Omniscript reverse transcriptase (Qiagen) with an oligo-dT primer. Primer pairs spanning the insertion site for RT-PCR on all five lines are as follows: AML1 117–138 5'gtgatggatgcgattggataga3', AML1 556-534rc 5'attgtggcttcagctggtaactt3'; AML2 86–109 5'tttgcttctccgattctcttcctt3', AML2 456-435rc 5'agcatcgggttcaacatcttcc3'; AML3 548–568 5'gtagcggaggaggtcttgaat3', AML3 1060-1039rc 5'tctccttgatctcgccataaac3'; AML4 1932–1955 5'aagcggtagtccatataaatcctc3', AML4 2944-2927rc 5'tcccctgaatccgaccat3'; AML5 104–124 5'cgtgatcatcgtcggtgttgg3', AML5 1047-1024rc 5'ctcgacgaatttgtgatgcctctta3'. Reactions were performed using Takara ExTaq and 35 cycles of 94°C for 30 sec, 58°C annealing (*AML1,2,4*) or 60°C annealing (*AML3,5*) for 30 sec, and 72°C for one minute.

Primer pairs amplifying a region 3' of the insertion site for RT-PCR on *AML1*, *AML2*, *AML3 *and *AML5 *insertion homozygotes are as follows: AML1 +1635 5'aggctctcgccgccctatta3', AML1 -2466 5'cgttgccaccttctcgctatt3'; AML2 +1779 5'accggggaacagtagtgaac3', AML2 -2107 5'ctgtcggcaagcatagaaag3'; AML3 +1756 5'tctggcctgctgctacaatgg3', AML3 -2326 5'cgccgacaagaagatgagaaaac3'; AML5 +1268 5'gcaacggcttccaacagtca3', AML5 -1869 5'acgaggcctaccattttcatacaa3'. All reactions were performed with a 59°C annealing temperature.

## Authors' contributions

GHA conceived of the study and performed all experimental manipulations. MRH provided guidance and arranged funding for the project. GHA and MRH drafted the manuscript, and both authors approve the final manuscript.
